# Quantifying the stability landscapes of psychological networks

**DOI:** 10.3758/s13428-025-02917-7

**Published:** 2026-02-20

**Authors:** Jingmeng Cui, Gabriela Lunansky, Anna Lichtwarck-Aschoff, Norman B. Mendoza, Fred Hasselman

**Affiliations:** 1 Faculty of Behavioural and Social Sciences, University of Groningen, Groningen, The Netherlands; 2 Amsterdam Public Health Institute, Amsterdam University Medical Center, Amsterdam, The Netherlands; 3 Department of Curriculum and Instruction, Faculty of Education and Human Development, The Education University of Hong Kong, 10 Lo Ping Road, New Territories, Tai Po, Hong Kong SAR; 4 Behavioural Science Institute, Radboud University, Nijmegen, The Netherlands

**Keywords:** Network models, System stability, Stability landscape, Ising model, Psychopathology

## Abstract

The network theory of psychopathology proposes that mental disorders can be represented as networks of interacting psychiatric symptoms. These direct symptom–symptom interactions can create a vicious cycle of symptom activation, pushing the network to a self-sustaining, dysfunctional phase of psychopathology: a mental disorder. Symptom network models can be estimated from empirical data through statistical models. Although simulation studies have established a relation between the structure of these symptom network models and the probability they end up in a self-sustaining dysfunctional phase, the general stability of the system is left implicit. The general stability includes both the stability of the dysfunctional phase and the stability of the healthy phase. In this paper, we present a novel method to quantify the stability landscapes of network models through stability landscapes. Our method is based on the Hamiltonian of the microstates of Ising models and can be used to show the stability of estimated Ising network models. Compared to simulation-based methods, our approach is computationally more efficient and quantifies the stability of all possible system states. Furthermore, we propose a set of stability metrics to quantify the stability of the healthy and dysfunctional phases and a bootstrapping method for range estimation of the stability metrics. To demonstrate the method’s utility, we apply it to an empirical data set and show how it can be used to compare the stability of phases between groups. The presented method is implemented in a freely available R package, *Isinglandr*.

**Supplementary Information:**

The online version contains supplementary material available at 10.3758/s13428-025-02917-7.

## Introduction

Over the past decades, there has been a growing interest in understanding psychopathology through the lens of network theory. This approach conceptualizes mental disorders not as isolated symptoms with a common underlying cause but as complex systems where symptoms interact with and influence one another (Cramer et al., 2016; Olthof et al., 2023b). In a network model of psychopathology, the analysis takes place at the level of interacting symptoms, where each symptom is represented as a node, and the connections (called edges) between nodes represent the relationships between symptoms (Borsboom, 2017). The interactions among symptoms are crucial for understanding the system’s overall behavior. For example, sleeping problems can lead to worrying thoughts, which in turn can increase fatigue and depressed mood (Cramer et al., 2016).

Individuals whose symptom networks have strong connections between symptoms may be more vulnerable to developing a mental disorder, as the activation of one symptom can trigger a cascade of others. Conversely, those with weaker connections may be more resilient. Often, the system is organized such that certain phases (i.e., qualitatively different patterns of behavior) emerge from the system, such as the healthy phase and the depressive phase (Cui et al., 2023). The stability of these phases can vary, explaining why some individuals show resilience while others experience persistent mental disorders (Olthof et al., 2020; Cramer et al., 2016).

One way to advance the understanding of psychopathology from a network perspective is to estimate statistical network models from empirical data. These models reveal the conditional associations between symptoms, controlling for all other variables (Borsboom et al., 2021; Bringmann et al., 2022; Epskamp et al., 2018). However, while these models provide insights into the relationships between symptoms, they do not directly indicate the stability of different system phases – meaning we cannot easily determine whether a group of people is more likely to be in a healthy or depressive phase based solely on their network structure. Understanding the stability of different phases is crucial both for clinical practice and for better theorizing mental disorders from a network perspective, yet methods for depicting phase stability in network models remain sparse.

To address this gap, researchers have used simulation-based approaches to predict the likely phase of a symptom network. For instance, studies have examined whether networks with strong associations among symptoms are more likely to result in a depressive phase (Cramer et al., 2016; Lunansky et al., 2025). However, these simulations often provide only a rough estimate and do not capture the overall stability of the entire system across different starting points. Moreover, the accuracy of these predictions can be influenced by random noise and other factors, making it challenging to draw definitive conclusions.

A useful way to conceptualize the stability of a symptom network is through the metaphor of a stability landscape. Imagine a ball rolling on an uneven landscape, where lower positions represent more stable states. The shape of the landscape determines where the ball (or system) is likely to settle. In this analogy, different basins on the landscape correspond to different psychological phases, such as the healthy phase and the depressed phase (Hayes & Andrews, 2020). The stability of these phases determines the direction in which the system is likely to evolve. For example, individuals characterized by a stability landscape with a deep and steep basin for the depressive phase are more likely to develop and remain in a depression, while those with a shallow basin for the healthy phase may be more resilient.

While the stability landscape metaphor has been frequently used in the literature of psychopathology, especially from the complex systems approach (Scheffer et al., 2018; van Bork et al., 2024; Hayes & Andrews, 2020; Olthof et al., 2023b), it has not been fully computed from estimated psychological networks. Recently, a method was proposed to calculate the stability landscape of formal dynamical models (Cui et al., 2023). In this paper, we will use those insights to introduce a novel method to compute the full stability landscape from networks estimated from cross-sectional data. This method allows us to infer the population-level stability of network states. Most network studies to date use cross-sectional data (Bringmann et al., 2022; Epskamp et al., 2018), however, efforts to estimate person-specific models based on multivariate timeseries data are pursued by many authors (Bringmann, 2021; Hulsmans et al., 2024; Mansueto et al., 2023; Olthof et al., 2023a; Wright & Woods, 2020). To draw inferences on the individual level, we would need to compute stability landscapes from longitudinal individual data. However, methods for estimating nonlinear networks from such data are still not fully developed, making it currently infeasible to compute individual landscapes.

In this paper, we move beyond the metaphor of stability landscapes and present, for the first time, a method for computing them from symptom networks. As a first step, we focus on cross-sectional data and stability landscapes for the group level. The paper is structured as follows: First, we introduce the concept of formal stability landscapes. Next, we explain how stability landscapes can be calculated from a cross-sectional Ising network model, which is particularly well-suited for demonstrating nonlinear dynamics and bistability (van Borkulo et al., 2014). We also propose a set of metrics to quantify phase stability, made available through the R-package *Isinglandr*, which can be applied to any psychological Ising network. Finally, we provide an empirical illustration of how stability landscapes can be used to compare different groups and discuss the main contributions and limitations of our method.

## Formalizing stability landscapes for psychological networks

Currently, there are numerous network psychometric models that can be applied to psychological systems to analyze the relationships among variables. The choice of model depends on the type of data being used. For instance, the Gaussian graphical model (GGM) is used for cross-sectional, continuous data, the vector autoregressive (VAR) model is used for longitudinal, continuous data, and the Ising network model is used for cross-sectional, binary data. GGM and VAR are both linear models and cannot represent bistable systems (Haslbeck et al., 2022). However, bistability is often assumed in theoretical formulations of psychopathology (Olthof et al., 2023b; Hayes & Andrews, 2020; Scheffer et al., 2018) and observed in clinical settings (Helmich et al., 2020; Miller, 2004; Tang & DeRubeis, 1999). The Ising network model, although not as widely used, is capable of showing the bistability of a system. Therefore, in this article, we use the Ising network model for illustration purposes, as in Lunansky et al. (2025); Cramer et al. (2016). A couple of other methods have been developed recently for modeling nonlinear relationships within the network framework (e.g., Slipetz et al., 2024; Hasselman & Bosman, 2020; Golino et al., 2022), but they are mainly designed for estimating the strength of nonlinear dependency, instead of modeling the exact form of the nonlinear relationship, which is required for estimating the potential function. Therefore, in this article, we primarily focus on the simpler case of the Ising model.

In an Ising network, the system state is represented by the activation state of each node, denoted as $\boldsymbol{a} = [{a_1}, {a_2}, ..., {a_n}]^T$. $\boldsymbol{a}$ is a vector with the same length as the number of nodes, and each element may take the value of 1 or 0, representing activity or inactivity. Therefore, the *i*-th element, ${a_i}$, represents the activation state of the *i*-th node, with 1 indicating an active state and 0 indicating an inactive state. In the context of psychological networks, active and inactive states correspond to the presence or absence of symptoms, respectively. The Ising network model consists of two types of parameters (van Borkulo et al., 2014): node thresholds and edge weights. Both classes of parameters can influence the tendency for a node to be activated. The threshold of a node reflects its inherent tendency for activation. When all other nodes are in the same state, increasing the threshold of a node makes it less likely to be activated. Edge weights represent the strength of the association between two nodes. For a given node, several other nodes may be connected to it through edges, and their values may make the target node more or less likely to be activated on top of its inherent tendency (represented by the threshold). The exact meaning of the edge weight varies depending on the representation of the node states (Haslbeck et al., 2021). In the current article, we adopt the standard representation used in most psychological literature, where a higher edge weight $w_{i,j}$ between node *i* and *j* indicates a higher probability that both nodes will be activated ($a_i = a_j = 1$) relative to all other possible node states (i.e., only one node is activated or neither node is activated). Therefore, decreasing the thresholds of nodes and increasing the edge weight of nodes makes it more likely that more nodes are activated.

The probability that a node is active, given the active states of all other nodes and the network parameters, can be calculated using the following formula (van Borkulo et al., 2014):

1 $$\begin{aligned}&P(a_i=1|{a_j, j=1,2,...,i-1, i+1, ..., N}) \nonumber \\&\quad =\frac{1}{1+e^{-\beta (m_i+\sum _{j=1,j\ne i}^N w_{i,j}a_j)}}, \end{aligned}$$

where $m_i$ is the threshold for the *i*-th node, $w_{i,j}$ is the edge weight between the *i*-th and *j*-th nodes ($w_{i,i}=0$), and $\beta$ represents the randomness of the system (typically set to 1). The thresholds for all nodes, denoted by $\boldsymbol{m}=[m_1, m_2,..., m_N]^T$, and the edge weights for each pair of nodes, denoted by $$\boldsymbol{W}=\left[ \begin{array}{ccc}w_{1,1} & \cdots & w_{1,N} \\ \vdots & \ddots & \vdots \\ w_{N,1} & \cdots & w_{N,N}\end{array}\right] $$, can be estimated using logistic regressions (van Borkulo et al., 2014). Together, these parameters fully describe an Ising network.

To formalize the stability landscape for psychological networks, draw upon the concept of the stability landscape from physics, which represents the potential energy in each possible state, and the movement tendency of the system is along the gradient of the landscape^1^:

2 $$\begin{aligned} \frac{\textrm{d}x(t)}{\textrm{d}t}=-\frac{\textrm{d}U(x)}{\textrm{d}x}, \end{aligned}$$

in which *x* is the state of the system, $\textrm{d}x/\textrm{d}t$ represent the speed of movement for the system, *U*(*x*) is the potential energy for a given state *x*, and $\textrm{d}U(x)/\textrm{d}x$ is how the potential energy increase or decrease along *x*, representing the steepness of the landscape. Intuitively speaking, this means the state of the system tends to go down to a lower place of the landscape, and when the landscape is steeper, it also moves faster. This is exactly what we mean by the ball-and-landscape analogy. Therefore, to search for a landscape definition for networks, we need to have a variable x to summarize the state of the system, as well as a function *U*(*x*) to represent the stability of the system in a given state.

**Fig. 1:**
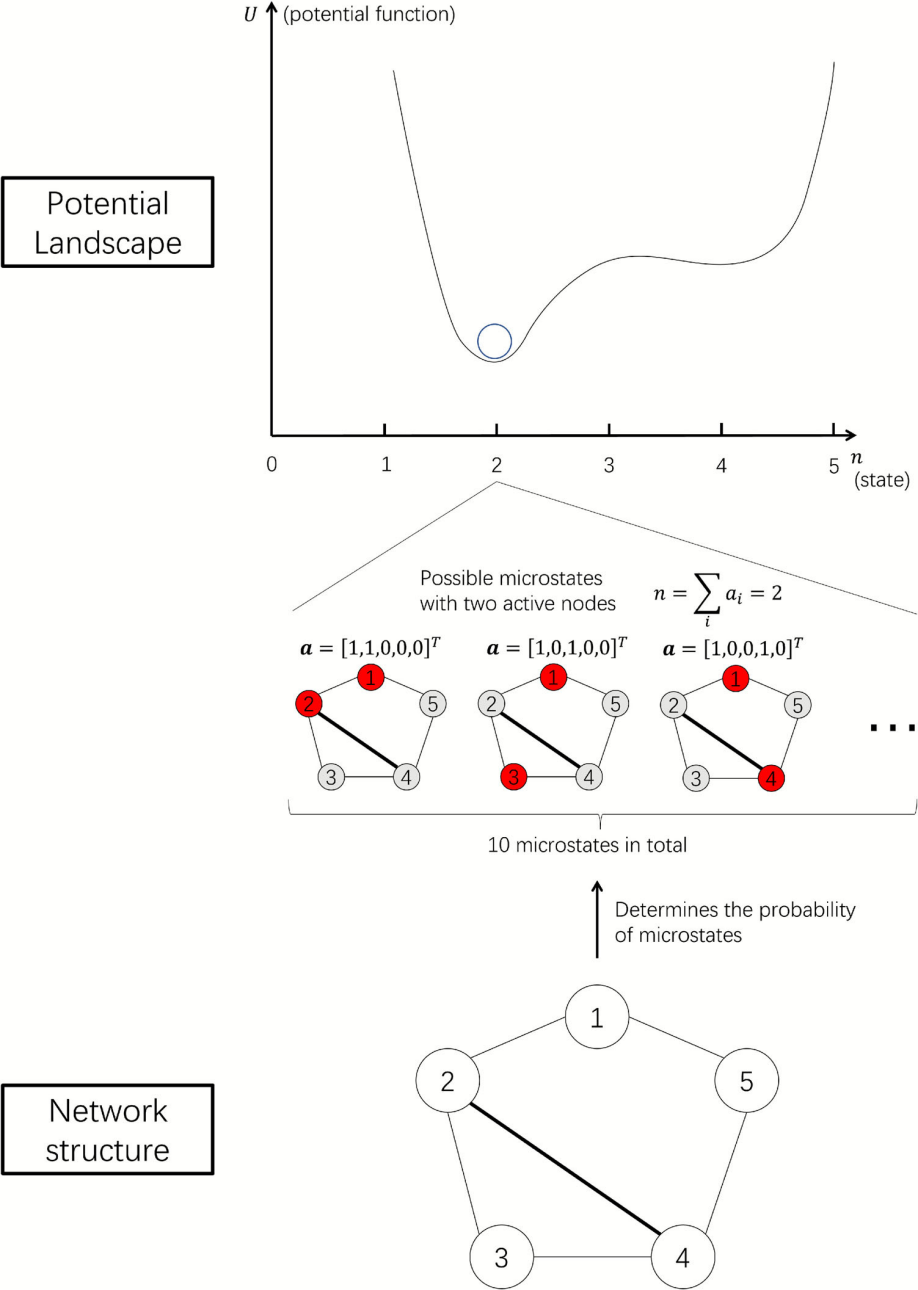
Diagram of the relationship between the network structure, the microstate, the state of the system, and the stability landscape. The network structure is arbitrarily chosen for illustration. *Red nodes* represent active nodes and *gray nodes* represent inactive nodes. The thickness of the edges represents the strength of the connection. See Eq. 3

For Ising networks, there is already an intrinsic energy measure for each possible activation state, namely, the Hamiltonian energy (Ising, 1925; also see Brusco et al., 2021 for theoretical discussions in the psychological literature). For a state of the network, $\boldsymbol{a}$, the Hamiltonian, *H*, is given by:

3 $$\begin{aligned} \begin{aligned} H(\boldsymbol{a})&=-\sum _{i = 1}^{n-1} \sum _{j=i+1}^{n} w_{i,j}a_ia_j -\sum _{i}a_im_i\\&=-\frac{1}{2}\boldsymbol{a}^T\boldsymbol{W}\boldsymbol{a}-\boldsymbol{a}^T\boldsymbol{m} \end{aligned} \end{aligned}$$

The probability that the system is in a state $\boldsymbol{a}$ is given by:

4 $$\begin{aligned} P(\boldsymbol{a})\propto e^{-\beta H(\boldsymbol{a})}. \end{aligned}$$

Can we use $H(\boldsymbol{a})$ to construct the stability landscape of the system? While theoretically possible, it is not practical due to the vast number of possible states ($2^N$), making it difficult to comprehensively list the Hamiltonian energy of each. However, in the case of psychopathology, the number of symptoms is often used as an indicator of the severity of the psychopathology, especially for diagnostic purposes (American Psychiatric Association, 2022). Therefore, as a solution to the vast number of possible states in the network model, we opt to use the number of active nodes,

5 $$\begin{aligned} n=\sum _{i=1}{a_i}, \end{aligned}$$

instead of the vector $\boldsymbol{a}$ as the representation of the system state. To clarify, in the remaining part of this article, we only use the term state for *x* (sometimes also referred to as *macrostate* in the literature), in contrast to the states represented by $\boldsymbol{a}$ as *microstates* (Dalege et al., 2018). Note that the state of the system represented by *n* needs to be a meaningful abstraction of the microstates. In other words, it should be theoretically meaningful to use the sum score to represent a characteristic of the target system. If the sum score of the nodes does not have a clear theoretical meaning, the summation of the nodes may not be appropriate.

Many potential microstate configurations may generate one state of the network. For example, if there are five nodes in a network, then there are $\textrm{C}_{5}^2=10$ possible microstates belonging to the state that two nodes are active (see Fig. 1 for several examples)^2^. To define the potential function of a state, we need to effectively summarize the information from its microstates. Here we introduce the generalized potential function by Wang et al. , (2008) (see also Cui et al., 2023). Wang’s landscape is defined by the steady-state distribution of the system, $P_\textrm{SS}$, which is a distribution of systems that does not change over time. If a large number of copies of an Ising network evolve for a long period of time, then ultimately, the distribution of the number of active nodes *n* will converge to $P_\textrm{SS}(n)$. Wang’s landscape function is defined as:

6 $$\begin{aligned} U(x) = -\ln {P_\textrm{SS}(x)}. \end{aligned}$$

At first glance, this definition of stability landscapes appears circular and equivalent to Eq. 4. However, Eq. 6 is more general because it can be used for other variables that represent the state of the system (here, the state *n* instead of the microstate of the system $\boldsymbol{a}$) while still providing meaningful results. For Ising networks, the steady-state distribution is the same as defined by Eq. 1,^3^. Hence, we can use Eq. 1 to calculate the steady-state distribution of the system analytically, without running simulations. Combining Eqs. 1, 5, and 6, we define the generalized stability landscape for the state variable *n* as follows^4^:

7 $$\begin{aligned} U(n)&= -\ln {P_\textrm{SS}(n)} \nonumber \\&= -\ln {\sum _{\boldsymbol{a}_i, \sum _{\boldsymbol{a}_i} = n} P(\boldsymbol{a}_i)}\nonumber \\&= -\ln {\sum _{\boldsymbol{a}_i, \sum _{\boldsymbol{a}_i} = n} e^{-\beta H(\boldsymbol{a}_i)}}, \end{aligned}$$

where $H(\boldsymbol{a})$ is defined by Eq. 3.

We developed Isinglandr, an R package that implements the methods presented in this article and produces the results reported here. The package is available from the Comprehensive R Archive Network (CRAN) at https://cran.r-project.org/package=Isinglandr, and the code used to generate the results is available on the Open Science Framework (OSF) repository at https://osf.io/y3kju/.

## Calculating the stability landscape of depression

In this section, we show how variations in the symptom network structure lead to different stability landscapes. This illustrates the relationship between the cross-sectional Ising network’s symptom network structures and the stability landscapes. Building on previous studies, we systematically adjust the parameters of an estimated cross-sectional baseline Ising network to demonstrate how the stability landscape changes with different network structures (Lunansky et al., 2025).

**Fig. 2:**
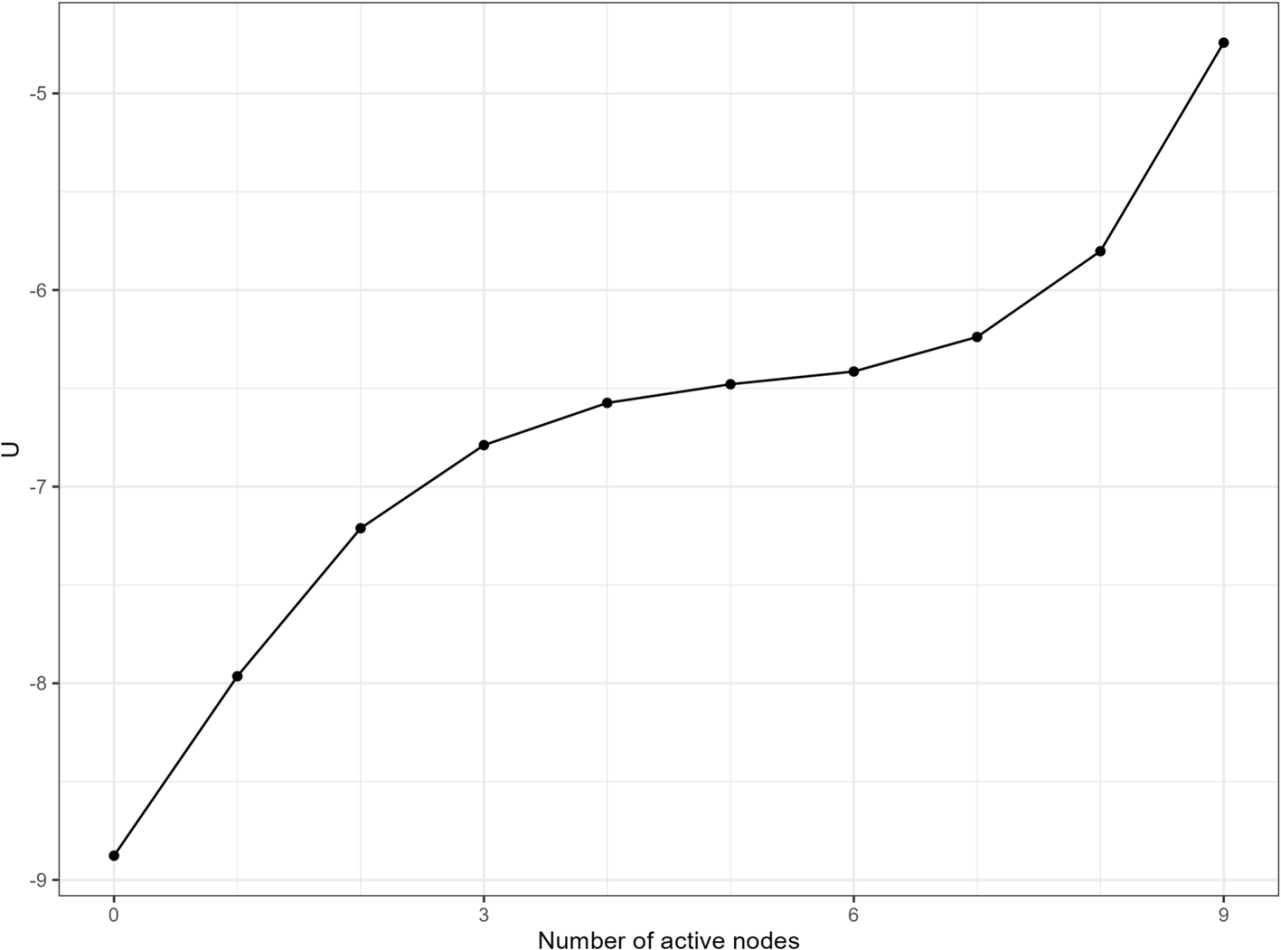
The landscape of the baseline network

**Fig. 3:**
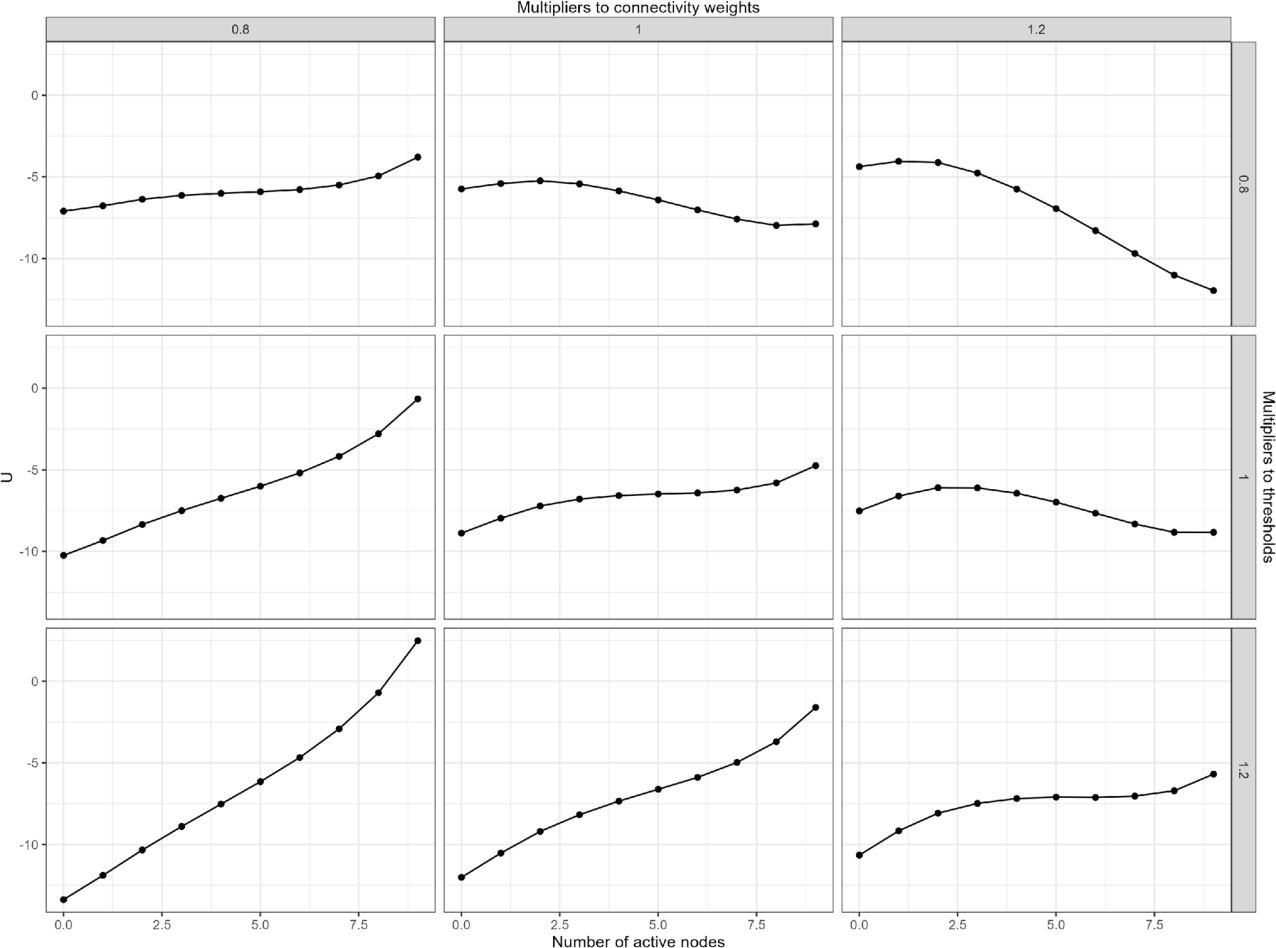
The landscapes of variations of the baseline network structure. Overall connectivity and threshold parameters of the baseline network are systematically multiplied by 0.8, 1, or 1.2, and the panels show the resulting stability landscapes

**Fig. 4:**
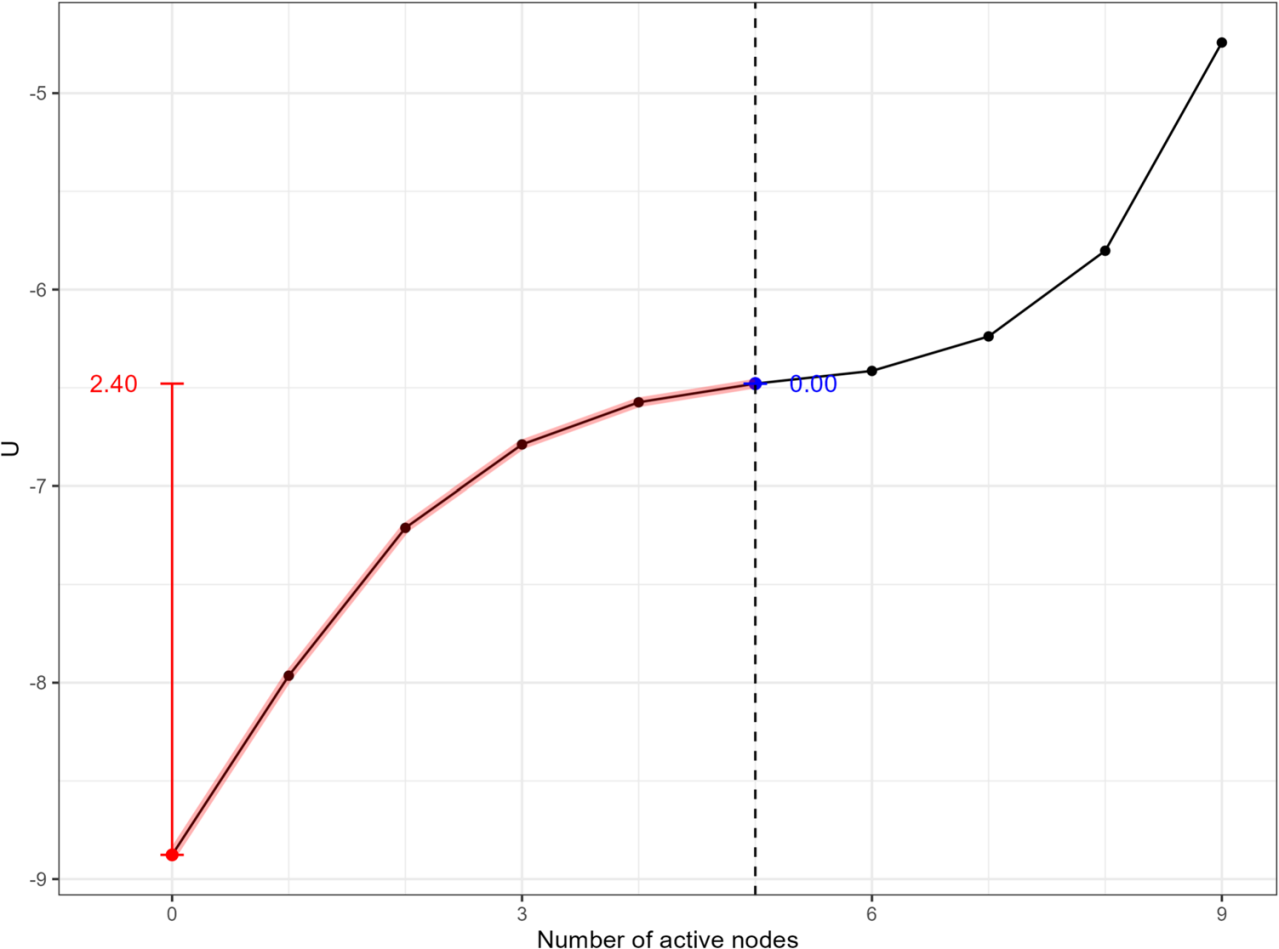
Stability metrics for the baseline network. The *red lines* indicate the range of the landscape function used for calculating the stability of the healthy phase, and the *blue lines* indicate the range of the landscape function used for calculating the stability of the depressive phase. The *vertical lines* represent the difference in the potential function *U* between two reference points. The *red* and *blue numbers* are the values of the stability metrics for the healthy and depressive phases, respectively

### Estimating the baseline model

The baseline network is estimated from data from the Virginia Adult Twin Study of Psychiatric and Substance Use Disorders (VATSPSUD; Kendler & Prescott, 1999). The data contain binary data on the presence/absence of nine depression symptoms from 8973 twins from the Mid-Atlantic Twin Registry (see the **Supplementary Materials** for a description of all nine depression symptoms). We received the estimated Ising network parameters from these data, which were estimated with the *psychonetrics* package in R (Epskamp, 2024).

The stability landscape of the baseline networks is shown in Fig. 2. The *x*-axis represents the number of active nodes (i.e., the system’s state), and the *y*-axis represents the generalized potential function of each state. Higher potential values indicate less stability for that state, on average within the sample. The potential function has a local minimum at $n = 0$ and a relatively flat region around $n = 6$, indicating two phases: the *healthy phase* (fewer active symptoms, $n=0$ to $n=5$) and the *depressive phase* (more active symptoms, $n=5$ to $n=9$). The lower potential at $n=0$ suggests that the healthy phase is more stable than the depressive phase, meaning that, although the system can reside in the depressive phase, it is likely to transition back to the healthy phase.

### Stability landscapes of varying symptom network structures

Alternative network structures were created by multiplying the baseline model’s connectivity and threshold parameters by constants (0.8, 1, or 1.2), allowing us to simulate increases, decreases, or no changes to these values. Figure 3 shows the resulting stability landscapes for these different parameter configurations. The *center panel* shows the stability landscape when both the threshold parameters and overall connectivity parameters are unchanged (multiplied by 1), matching the baseline stability landscape in Fig. 2.

**Fig. 5:**
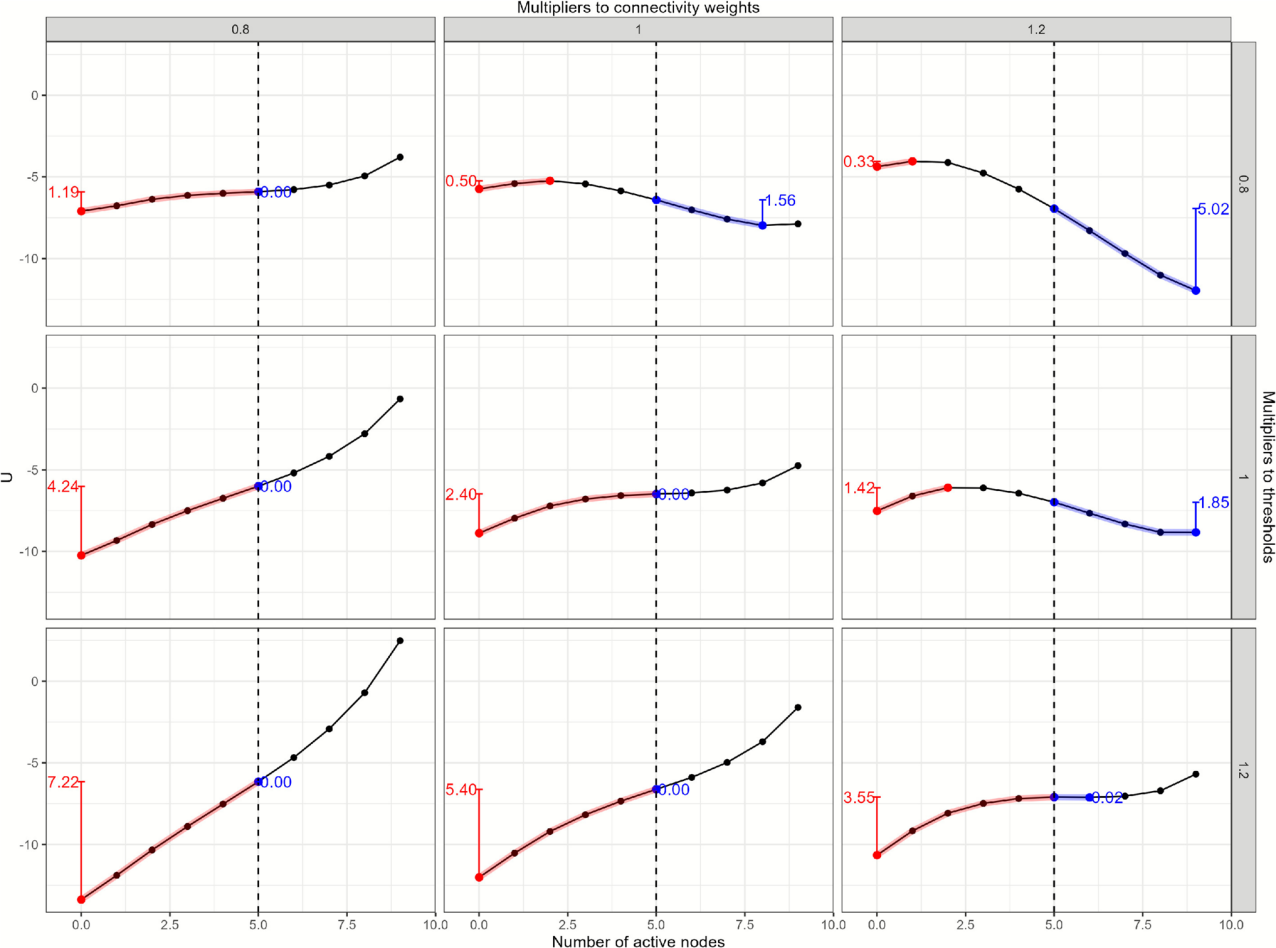
Stability metrics for the landscapes of variations of the baseline network structure. The *red lines* indicate the range of the landscape function used for calculating the stability of the healthy phase, and the *blue lines* indicate the range of the landscape function used for calculating the stability of the depressive phase. The *vertical lines* represent the difference in the potential function *U* between two reference points. The *red* and *blue numbers* are the values of the stability metrics for the healthy and depressive phases, respectively

Overall, we observe that weaker connectivity (multiplied by 0.8) and stronger thresholds (multiplied by 1.2) lead to fewer activated nodes and a more stable healthy phase. This is evident in the lower-left panel, where the stability landscape shows only one phase with a local minimum at $n=0$, indicating that the corresponding network tends toward having fewer active symptoms. Conversely, increased connectivity (multiplied by 1.2) and weaker thresholds (multiplied by 0.8) result in more activated symptoms, enhancing the stability of the depressive phase. This scenario is illustrated in the upper-right panel, where the depressive phase becomes more stable, with a local minimum at $n=9$.

When changes in connectivity and threshold values offset each other, the effects on the stability landscape may cancel out, as shown in the upper-left and lower-right panels, where the stability landscapes remain similar to the baseline.

For readers interested in further exploring how parameter cha-nges influence system stability, the *Isinglandr* package’s Shiny app (Isinglandr::shiny_Isingland_MDD()) al-lows for interactive parameter adjustments and visualizations.

### Stability indicator metrics

To better interpret the stability landscapes, we propose a set of metrics to quantify the stability of the healthy and depressive phases for different network structures. The stability landscape provides a quantitative representation of the stability of the network states, serving as a basis for describing the relative stability of these phases. Previous studies have suggested using barrier height as a relative stability measure of psychological phases (Cui et al., 2023), but this metric is undefined when there is only one stable phase in the landscape, and it does not account for the clinical significance of the number of symptoms.

To address these limitations, we use the clinical cutoff of depression (in this example, five active symptoms out of nine nodes as in DSM-5-tr, American Psychiatric Association, 2022) to demarcate the healthy and depressive phases on the stability landscape. Specifically, the stability of the healthy phase is calculated based on the left portion of the potential function (states with fewer than five active symptoms), and the stability of the depressive phase is calculated from the right portion (five or more active symptoms).

Within the left portion, the difference between the leftmost local minimum and the maximum value to the right of that local minimum is used as the stability of the healthy phase, and the stability of the depressive phase is defined symmetrically. This approach ensures consistency with clinical definitions of depressive diagnosis and severity. The resulting stability metrics are shown in Figs.  4 and 5. The figures illustrate how different parameterizations affect the stability of each phase, with the healthy phase becoming less stable and the depressive phase more stable as network connectivity increases and thresholds decrease.

To capture the overall stability of both phases, we calculate the stability difference by subtracting the depressive-phase stability metric from the healthy-phase stability metric. A positive stability difference indicates that the healthy phase is more stable than the depressive phase, with a larger absolute value reflecting a greater relative stability of the healthy phase. In contrast, a negative stability difference suggests that the depressive phase is more stable, with larger values indicating a stronger tendency in the sample towards the depressive phase. The stability differences for all the landscapes of variations of the baseline network structure are shown in Table 1.

#### Bootstrapping for stability metric uncertainty

For empirical datasets, we can also estimate the uncertainty in the stability metrics using bootstrapping. The bootstrapping method has been widely used in various psychometric contexts for estimating the uncertainty of parameters (e.g., Epskamp et al., 2018; Mallinckrodt et al., 2006; Wright et al., 2011). It resamples participants with replacement from the original dataset many times, estimates the parameters from the resampled datasets, and uses those parameter values to obtain the range estimation of the parameter. Compared with parametric methods, the bootstrapping method does not require specific knowledge about the parameter distribution, thus is suitable for the stability metrics. The resampling process should be operated from the original dataset instead of the network parameters. Therefore, we will only explain the method here and leave concrete examples for the next section, where the original dataset used for network estimation is available.

In the Isinglandr package, we use the *boot* (Davison & Hinkley, 1997) and *boot.pval* (Thulin, 2023) packages to estimate the standard error, confidence interval, *p* values, as well as the significance of the stability difference. We apply the bias-corrected and accelerated (BCa) method as the bootstrap distribution of the stability difference is often skewed. Previous research has shown that the BCa method performs the best in such cases (Puth et al., 2015).

## Using stability landscapes to compare groups

Network analysis is a powerful tool that can provide an overview of the specific interactions between variables in a population of interest, making it highly suitable for comparisons between groups (e.g., males and females; Burger et al., 2023). Network comparison offers a comprehensive way to gain insight into group differences, such as whether specific edges between nodes vary across populations (e.g., gender differences in psychopathology networks; Kendler et al., 2022, or differences in stress disorder networks between young adults and adolescents; Sun and Zhou, 2023). In the context of psychopathology, group-level network comparisons have been used to understand whether different groups, based on, for example, age or gender, may need different clinical treatments (Lee & Hu, 2022). In this section, we demonstrate how stability landscapes can be used for group-level network comparisons with an empirical illustration, and we explain how this approach differs from a widely used *Network Comparison Test* (NCT, van Borkulo, 2023).

The NCT is a statistical test that compares network structures between groups using resampling-based permutation (van Borkulo, 2023). The test checks for significant differences in the (1) overall network structure, (2) global connectivity of the networks, and, if global differences are detected, (3) differences in strength between specific edges. The test is implemented in the software package *NetworkComparisonTest* (van Borkulo, 2023) within the R-environment (R Core Team, 2024). The test can be used for various network models, including the Ising network model.

### Empirical illustration

Here, we give an empirical illustration in which we compare psychopathology symptom networks between individuals with low and normal-to-high resilience. We show how to compare the stability landscapes and compute the stability difference between the groups, in addition to applying the Network Comparison Test (van Borkulo, 2023).

**Table 1:** Stability differences for the landscapes of variations of the baseline network structure. Higher values indicate that the healthy phase is more stable compared with the depressive phase

Multipliers to thresholds	Multipliers to connectivity weights	Stability difference
0.80	0.80	1.09
0.80	1.00	-1.06
0.80	1.20	-4.69
1.00	0.80	3.49
1.00	1.00	2.30
1.00	1.20	-0.43
1.20	0.80	5.85
1.20	1.00	4.67
1.20	1.20	3.45

### Methods

**Data** The data contains assessments of coronavirus anxiety symptoms using the Coronavirus Pandemic Anxiety Scale (CPAS-11, Bernardo et al., 2022) and the Brief Resilience Scale (BRS, Smith et al., 2008) in 2436 participants. The data was collected online during the outbreak of the Delta variant of SARS-CoV-2 in the Philippines (see Mendoza et al., 2022 and Dizon et al., 2023 for more details on the data collection procedure).

**Fig. 6:**
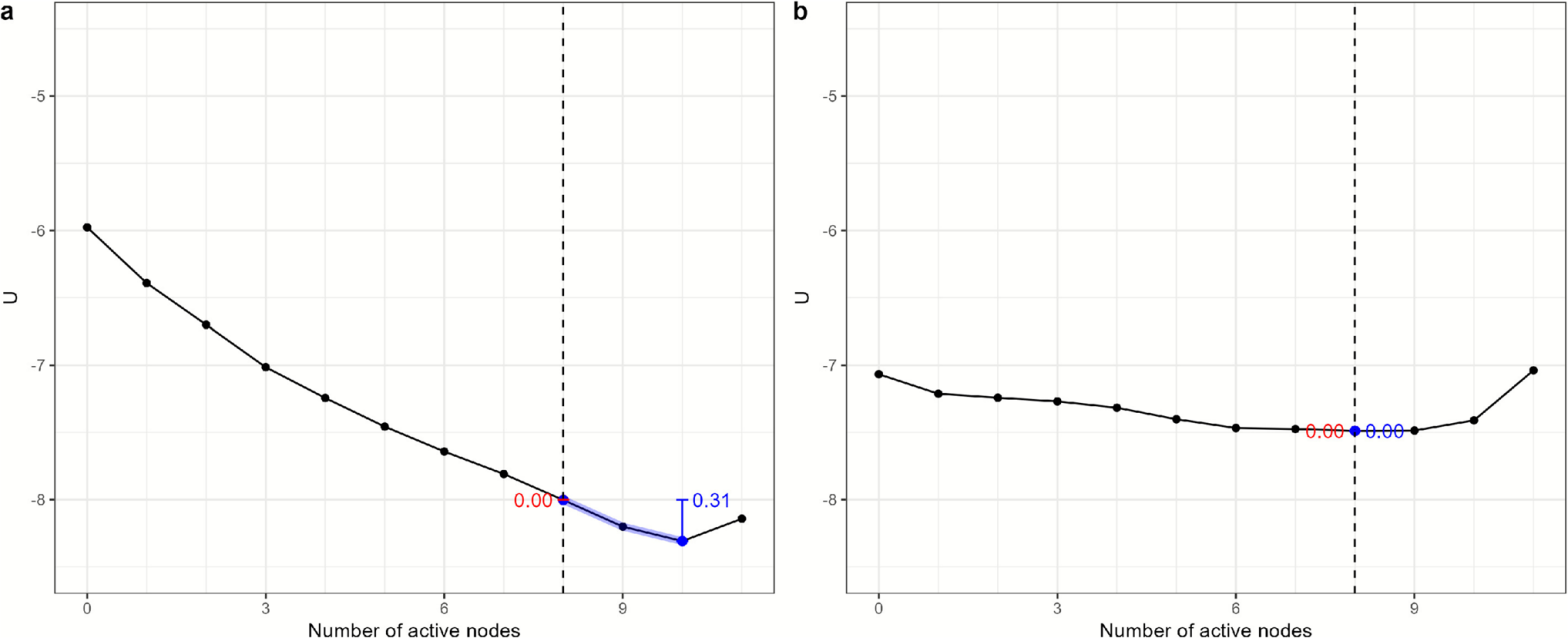
The landscapes of two groups. The cutoff value for the anxious phase is 8 symptoms or more (*dotted vertical line*). Panel **a** shows the stability landscape for the low-resilience group. The stable point is on ten actively present symptoms, which fall within the anxious phase. Panel **b** shows the stability landscape for the normal-to-high-resilience group. The stability landscape shows a much wider basin with a stable point that falls within the healthy phase

#### Networks

We split the group based on their BRS scores according to the cutoff values as reported by Smith et al. (2013): low resilience (BRS < 3, *n* = 836) versus normal-to-high resilience (BRS $\ge$ 3, *n* = 1551). When comparing groups, it is best that sample sizes are equal to ensure that differences in the estimated networks are not due to differences in power (van Borkulo, 2023). Therefore, we randomly sampled 836 participants from the normal-to-high resilience group. In this way, both networks are estimated from the same number of participants.

We then estimated the Ising network models of the coronavirus anxiety symptoms (CPAS-11) for these groups using the psychonetrics package in R (Epskamp, 2024). To do so, we first binarized the CPAS-11 scores as the Ising network model is estimated from binary data (van Borkulo et al., 2014). The CPAS-11 items range from 0 (no symptom presence at all) to 3 (symptom presence nearly every day). We chose to binarize on symptom absence versus some symptom presence. This means that all CPAS-11 values $\ge$ 1 are recoded into 1. The cutoff value of CPAS-11 is 15 for the 0–3 scale (Bernardo et al., 2022). This corresponds to eight active nodes for the binarized scale (see the **Supplementary Materials** for details) and we will use this value for calculating the stability metrics. This means that the healthy phase consists of a sum score of actively present symptoms below 8, and the anxious phase starts at 8 actively present symptoms or more. Note that we did not include the resilience factors (BRS) in the networks, as these variables were used to split the groups. Including variables into the networks that were initially used to split the groups could potentially introduce bias (de Ron et al., 2021; Haslbeck et al., 2022).

#### Comparison of the two networks

From these networks, we (1) compute the stability landscapes as described in the previous section, (2) compute the stability difference, and (3) apply the NCT to test for significant differences in global connectivity and overall structure between the two symptom networks of resilience groups (van Borkulo, 2023).

### Results

Figure 6 shows the results for the stability landscapes computed from the symptom network of low-resilience participants (left; panel a) and normal-to-high-resilience participants (right; panel b). The stability landscape of the low-resilience group follows a steep line towards a stable point of 10 actively present symptoms. This is above the cutoff value of eight symptoms or higher, meaning that the anxious phase is stable for the low-resilience group. The stability metric for the healthy phase is 0.00, and the stability metric for the anxious phase is 0.31. However, the landscape for the normal-to-high-resilience group shows a much wider basin with stability around eight actively present symptoms, which falls at the cutoff value of the anxious phase. Thus, the healthy phase is as stable as the anxious phase for the normal-to-high-resilience group. The stability metric for the healthy phase is 0.00, and the stability metric for the anxious phase is 0.00. The stability difference for the low-resilience group is -0.31 (*SE* = 0.06, 95% CI [-0.42, -0.19], *p* = .002) and for the normal-to-high resilience group is 0.00 (*SE* = 0.03, 95% CI [-0.10, 0.03], *p* = .567). The difference between the two groups is 0.31 (*SE* = 0.07, 95% CI [0.17, 0.43], *p* = .002).

**Fig. 7:**
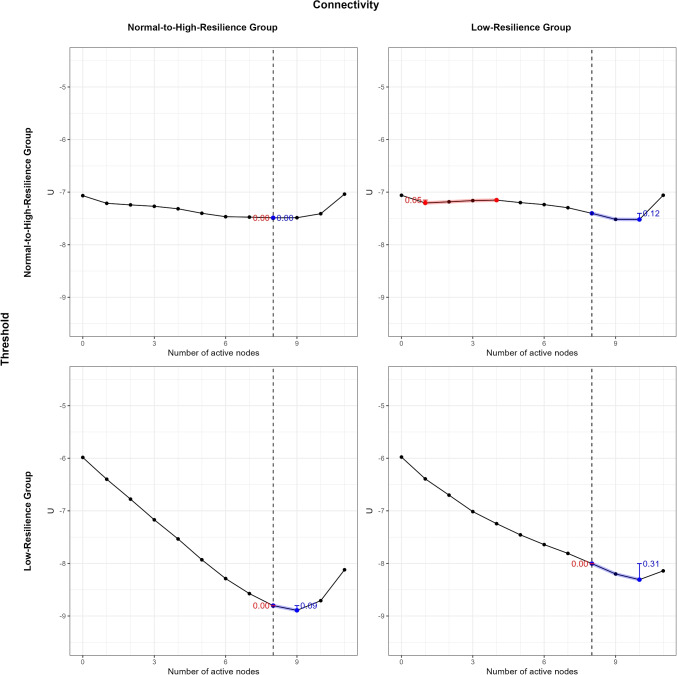
The landscapes for all four combinations of parameters from the normal-to-high- and low-resilience groups. Columns correspond to connectivity parameters from the normal-to-high-resilience group (first column) and the low-resilience group (second column), and rows correspond to threshold parameters from the normal-to-highresilience group (first row) and the low-resilience group (second row)

The NCT reported no significant differences in overall structure (*M* = 0.51, *p* = .98) nor global connectivity (*S* = 0.85, *p* = .69). As the test found no global differences, we did not look further into potential local differences between specific edges.

The difference in the stability landscape also implies differences in symptom levels between the two groups. To confirm this, we performed a post hoc permutation test (*B* = 10,000; two-sided) on the symptom counts. Group sizes were equalized by random subsampling from the larger group. The low-resilience group had, on average, 1.95 more active symptoms than the normal-to-high-resilience group (*p* < 0.001). A Welch’s *t* test yielded similar results (*t* = 12.80, *df* = 1634.7, *p* < .001). While the NCT indicated no significant connectivity differences, these results confirm a large and statistically significant mean difference in symptom counts between groups.

The stability landscapes for Ising networks are calculated from both the connectivity parameters and threshold parameters. To determine whether thresholds or connectivity parameters primarily drive group differences, we computed stability landscapes for all four parameter combinations from the normal-to-high- and low-resilience groups. This allowed us to systematically compare the contributions of thresholds and connectivity to the observed differences. The results (Fig. 7) clearly show that differences in stability landscapes are primarily driven by the threshold parameters from the normal-to-high- and low-resilience groups rather than by the connectivity parameters.

### Conclusion

In this section, we demonstrated the use of stability landscapes of networks to compare groups and provided an empirical illustration that can serve as an example for future research. We found that the anxious phase was stable for the low-resilience group, while the healthy phase was stable for the normal-to-high-resilience group. However, the NCT did not reveal differences between the two estimated networks. Thus, comparing networks of two groups based on their underlying stability landscape may yield different results than the NCT. This is because the stability landscapes are computed based on Ising networks, which consist of both connectivity and threshold parameters. The NCT can be used on Ising networks, but it only compares the networks on their structure (i.e., edge weights). There may be differences between groups that are captured within the threshold parameters but not considered by the NCT. By constructing stability landscapes using the combinations of parameters from different groups, we can see that the stability difference between the two groups mainly comes from the difference in thresholds instead of connectivity parameters. This also explains the potential differences between the NCT and stability landscapes. Therefore, we recommend using both to complement each other.

## General discussion

In this article, we proposed a novel method to quantify the stability of Ising networks in psychology using the generalized stability landscape function. The generalized stability landscape function represents the stability of the network’s different states (i.e., the sum of actively present nodes). The lower the potential, the more likely that the network will end up in that state – just like a ball on a landscape that tends to roll down to a lower place. The main purpose of the method is to provide a quantitative, intuitive representation of the stability landscape of psychological networks. Instead of only studying the stability of the current phases of the system using simulations (Lunansky et al., 2025), the method presented in this paper computes the full stability landscape of the system as a whole. This allows us to understand the system’s phases and their relative stability, as well as to compare the system’s stability across groups.

We connected the computation of stability landscapes with the network theory of psychopathology (Borsboom, 2017) and demonstrated how the potential function can be used to quantify the stability landscape of psychopathology networks estimated from cross-sectional data that consist of Major Depressive Disorder (MDD) symptoms. As an illustration, we showed how variations of the depression symptoms network lead to different stability landscapes. Furthermore, we proposed a set of stability metrics from the landscape function.

Finally, we used an empirical example to demonstrate how different stability landscapes can be empirically estimated from cross-sectional data. We estimated networks of PTSD symptoms in two groups with different levels of resilience, as measured by the Brief Resilience Scale. Afterward, we estimated the corresponding stability landscapes and showed how group-level differences in resilience indeed indicated differences in stability landscapes from symptom networks. By also considering the threshold parameters of the networks, comparing groups by their stability landscapes provides an added value next to existing network comparison methods, such as the *Network Comparison Test* (van Borkulo, 2023). From the landscapes for different combinations of the thresholds and connectivity parameters from two groups, we can confirm that, in this empirical example, the stability landscape differences were primarily driven by thresholds rather than connectivity parameters. While such results may appear trivial, the network theory of psychopathology has emphasized connectivity as the main driver of system behavior (Borsboom, 2017), and applied network comparisons of cross-sectional data often report only connectivity-based metrics, such as structural characteristics of the network and network comparisons using the NCT. Recent papers have argued for more attention to threshold-based comparisons in the network literature (Airaksinen et al., 2020; Elovainio et al., 2021). By integrating both thresholds and connectivity parameters, the stability landscape approach can detect stability differences that other methods would miss. As such, the proposed method is a meaningful complement to the current network analysis toolbox.

The current method holds several assumptions that we would like to clarify. First, the method uses sum scores as a representation of the network. For example, using the number of depression symptoms as an indicator of depression severity. As such, it does not differentiate between specific active symptoms. However, specific symptoms can have different thresholds for activation; for example, depressed mood may be more easily activated than suicidal ideation. However, once active, both nodes contribute equally to the symptom severity. We believe this approach is an acceptable simplification for symptom networks of several specific disorders. However, this assumption may not necessarily hold for networks with more differentiated symptoms. For example, networks consisting of combinations of depression symptoms and anxiety symptoms, bipolar disorder symptoms, or networks with nodes that extend beyond mere symptoms, such as risk or protective factors.

It is also important to note that we used the Ising network models estimated from cross-sectional group data to represent psychological systems and construct landscapes. This means that conclusions can only be made at the group level. If one is interested in interpretations on the individual level, cross-sectional models are only meaningful when the group is homogeneous enough, or in other words, with high ergodicity (Fisher et al., 2018; Molenaar, 2004). Therefore, all the limitations of cross-sectional models for individual inferences will also apply to the landscapes constructed therefrom. One possibility to overcome this limitation would be the development of idiographic methods to estimate Ising models from longitudinal, individual data, which are currently lacking. A second possibility would be to compute stability landscapes from other types of network models, which are estimated from individual data, such as vector autoregression (VAR) models. These models are estimated from within-person variance and can be estimated from ordinal or continuous data, instead of binary data in the Ising model. One example that is moving in this direction is given by Hoekstra et al. (2024). However, these models are linear and cannot account for multistability, which is only possible in nonlinear models (Haslbeck & Ryan, 2022; Haslbeck et al., 2022). However, even if methods to compute stability landscapes from idiographic network models were developed in future research, it would not be straightforward to collect the necessary data from an individual to compute their stability landscape. The data collection period would need to span a long period of time with enough variability to assess not only the individual’s current phase but also potential alternative phases that are captured by the landscape. An alternative way forward would be to combine cross-sectional and idiographic approaches. Cross-sectional methods, such as the proposed approach in this paper, could identify typical networks and stability landscapes that are characteristic of averaged healthy or unhealthy phases. Idiographic data collection would then be used to identify the type of network and stability landscape that best describes specific individuals. Currently, it remains an open question how this could be done exactly, but we believe it would make a highly interesting novel research line to investigate.

We see several topics for future research that could further the presented approach. First, instead of defining a clinical cutoff value between a healthy or dysfunctional state beforehand, one could, in theory, use the *shape* of the stability landscape to this aim. For example, if one has data from a general population and the stability landscape shows bistability, the area that separates the two stable states could contain a meaningful cut-off value that separates the healthy sample from the clinical sample. The simulations with adjusted empirical networks in the current paper did not find such bistability, but future research could focus on finding an example of a dataset that leads to a bistable stability landscape and determine the optimal method to use the shape of this landscape for diagnostic purposes.

Second, the empirical validation of the presented method is, of course, a pressing topic for future research. An interesting topic would be further investigating the relationship between the stability metrics and specific network structures. In the current study, we found that landscape differences are mainly associated with node thresholds rather than network connectivity strength. This may be different in another research context. Researchers may also look into the relationship between the stability metrics and other variables, for example, treatment outcome. In this way, we can gain more understanding of the meaning of the system’s stability and its practical implications.

Finally, we used symptom networks as illustrations, but the use of this method is not restricted to symptom networks. In principle, the same method can also be used for any other Ising networks for which the aforementioned two assumptions hold. For example, to understand how attitudes (feelings, beliefs, and behaviors) evolve. The Causal Attitude Network (CAN) model uses the Ising network model to explain how people move from negative attitudes about something or someone towards a more positive attitude, or vice versa (Dalege et al., 2017). It could be interesting to compute the stability landscapes of attitude networks to understand how stable positive or negative attitudes about, for example, politicians are Dalege et al. (2017). Similarly, while we used psychonetrics (Epskamp, 2024) to estimate Ising network models throughout the current article, the landscape construction method we propose is not exclusive to this estimation technique. Alternative approaches for estimating Ising network models have been suggested recently, including nonregularized and multivariate estimations (Brusco et al., 2023), as well as methods for correcting selection bias (Boot et al., 2023). Despite the differences in estimation methods, as long as the resulting network model remains an Ising network, the structure of the Hamiltonian remains consistent. Consequently, Ising networks derived from other estimation techniques can be employed for landscape construction using the same methodology outlined in this article. With this proposed method, we are one step closer to understanding the complexity and dynamics of psychological systems.

## Supplementary Information

Below is the link to the electronic supplementary material.

Supplementary file 1 (pdf 529 KB)

## Data Availability

The data used in this study are publicly available at the following OSF repository: https://osf.io/y3kju/.

## Footnotes

1 Under zero-inertia condition; the constant coefficient is omitted.

2 The number of microstates belonging to a state is termed as the *multiplicity* of the state.

3 Strictly speaking, it is the other way around. The Ising network model itself does not specify how the system evolves over time. Therefore, the steady-state distribution of common simulation algorithms for Ising systems all converge to the possibility distribution defined by Eq. 1 (e.g., the Glauber dynamics, Glauber, 1963).

4 Constants are omitted for brevity.

## Code Availability

The code necessary to reproduce all analyses and results presented in this article is publicly available at the following OSF repository: https://osf.io/y3kju/.

## Declarations

### Open practices statement

Data and analysis code are available at https://osf.io/y3kju/. None of the reported studies were preregistered. The preprint of this manuscript has been released at PsyArXiv: https://doi.org/10.31234/osf.io/nd8zc.
