# Supplementary material for: Quantifying the stability landscapes of psychological networks
Source: Behav Res Methods. 2026 Feb 20;58(3):68. doi: 10.3758/s13428-025-02917-7 (PMC12923465; doi:10.3758/s13428-025-02917-7)
Supplement: Supplementary file 1 — (pdf 529 KB) [file 13428_2025_2917_MOESM1_ESM.pdf]

Supplementary Materials

S1. Description of the nine symptom measures (DSM IV)

Table S1. MDD Symptoms

**DSM Symptom criteria**
(1) Depressed most of the day, nearly every day
(2) Markedly diminished interest or pleasure in all, or almost all, activities most of the day, nearly every day
(3) Significant weight loss when not dieting or weight gain (e.g., change of more than 5% of body weight in a month), or decrease or increase in appetite nearly every day
(4) Insomnia or hypersomnia nearly every day
(5) Psychomotor agitation or retardation nearly every day
(6) Fatigue or loss of energy nearly every day
(7) Feelings of worthlessness or excessive or inappropriate guilt nearly every day
(8) Diminished ability to think or concentrate, or indecisiveness, nearly every day
(9) Recurrent thoughts of death, recurrent suicidal ideation without a specific plan, or a suicide attempt or a specific plan for committing suicide

S2. Determining the cutoff value for the binarized CPAS-11 scale

In order to estimate an Ising network, a crucial step involves binarizing all the responses, whereby any non-zero response is considered "active." For the original CPAS-11 questionnaire, the predefined cutoff value is set at 15. To determine the ideal cutoff value for the binarized data, one that closely aligns with the original data, we employ the algorithm provided by the OptimalCutpoints package (López-Ratón et al., 2014) with Youden’s index (Youden, 1950), which maximizes the sum of sensitivity and specificity. The result suggests that the optimal cutoff value for the binarized scale should be 8. See Figure S1 for the distribution relationship between the raw scores and the binarized scores.

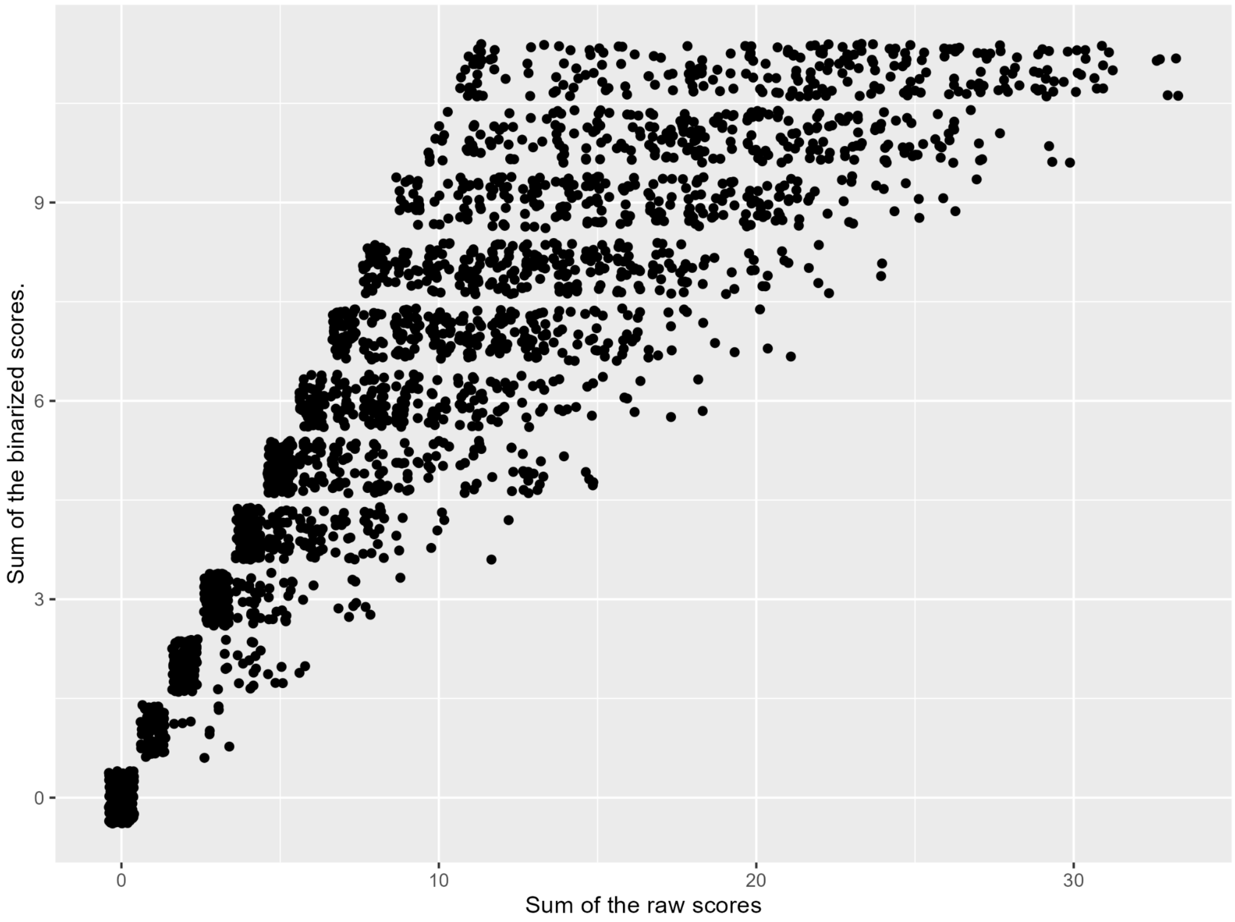

Figure S1. The distribution relationship between the raw scores and the binarized scores.

S3. Landscapes for networks with the same connectivity matrix

In Figure S2, we show the stability landscapes using the two networks with different connectivity parameters but using the threshold parameters from the low resilience group in both models. In this case, the landscapes were much more similar.

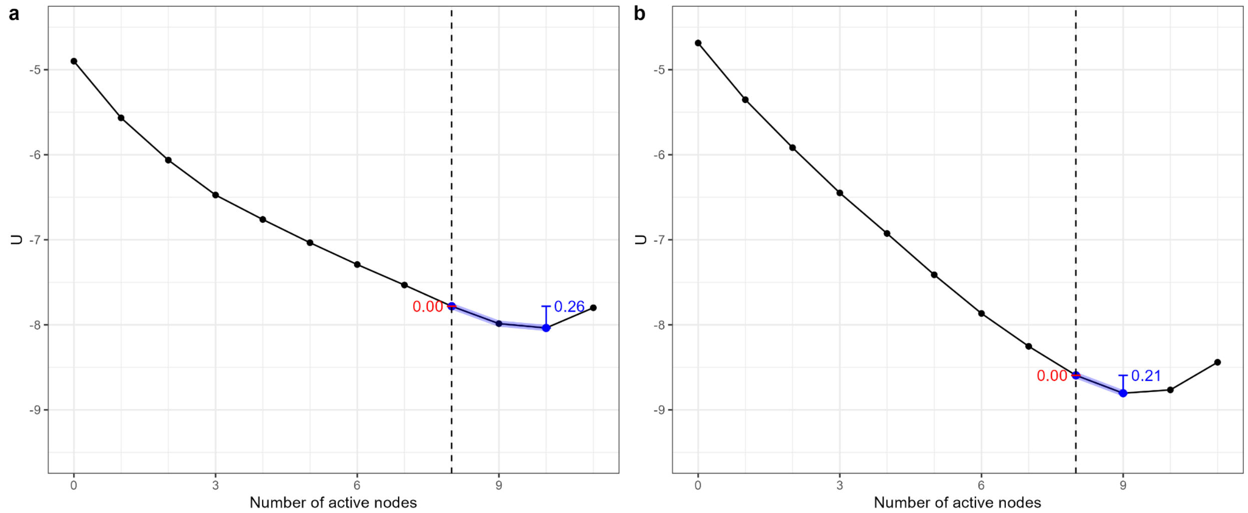

Figure S2. The landscapes for (a) the low resilience group, and (b) the network with the connectivity parameters from the high resilience group and the threshold parameters from the low resilience group.

References

López-Ratón, M., Rodríguez-Álvarez, M. X., Suárez, C. C., & Sampedro, F. G. (2014). OptimalCutpoints: An R package for selecting optimal cutpoints in diagnostic tests. *Journal of Statistical Software*, *61*(8), 1–36. https://doi.org/10.18637/jss.v061.i08

Youden, W. J. (1950). Index for rating diagnostic tests. *Cancer*, *3*(1), 32–35. https://doi.org/10.1002/1097-0142(1950)3:1<32::AID-CNCR2820030106>3.0.CO;2-3
